# The serotonin 5-HT_2C_ receptor and the non-addictive nature of classic hallucinogens

**DOI:** 10.1177/0269881116677104

**Published:** 2016-11-15

**Authors:** Clinton E Canal, Kevin S Murnane

**Affiliations:** 1 Center for Drug Discovery, Department of Pharmaceutical Sciences, Northeastern University, Boston, USA; 2 Department of Pharmaceutical Sciences, Mercer University College of Pharmacy, Mercer University Health Sciences Center, Atlanta, USA

**Keywords:** Hallucinogens, 5-HT_2C_, cocaine, Kv1, addiction

## Abstract

Classic hallucinogens share pharmacology as serotonin 5-HT_2A_, 5-HT_2B_, and 5-HT_2C_ receptor agonists. Unique among most other Schedule 1 drugs, they are generally non-addictive and can be effective tools in the treatment of addiction. Mechanisms underlying these attributes are largely unknown. However, many preclinical studies show that 5-HT_2C_ agonists counteract the addictive effects of drugs from several classes, suggesting this pharmacological property of classic hallucinogens may be significant. Drawing from a comprehensive analysis of preclinical behavior, neuroanatomy, and neurochemistry studies, this review builds rationale for this hypothesis, and also proposes a testable, neurobiological framework. 5-HT_2C_ agonists work, in part, by modulating dopamine neuron activity in the ventral tegmental area—nucleus accumbens (NAc) reward pathway. We argue that activation of 5-HT_2C_ receptors on NAc shell, GABAergic, medium spiny neurons inhibits potassium Kv1.x channels, thereby enhancing inhibitory activity via intrinsic mechanisms. Together with experiments that show that addictive drugs, such as cocaine, potentiate Kv1.x channels, thereby suppressing NAc shell GABAergic activity, this hypothesis provides a mechanism by which classic hallucinogen-mediated stimulation of 5-HT_2C_ receptors could thwart addiction. It also provides a potential reason for the non-addictive nature of classic hallucinogens.

## Classic hallucinogens are serotonin 5-HT_2_ receptor agonists

Classic hallucinogens (CH) are powerful psychoactive substances that are categorized into two broad chemotype classes: indoleamines (including tryptamines and ergotamines) and phenylalkylamines (including phenethylamines and phenylisopropylamines; Halberstadt, 2015). Prototypical CH representative of each subclass include psilocybin, a tryptamine found in several genera of mushrooms (Stamets, 1996); lysergic acid diethylamide (LSD), an ergotamine originally derived from ergot fungi (Hofmann, 1970); mescaline, a phenethylamine found in peyote (Heffter, 1898) and other cacti; and 2,5-dimethoxy-4-bromoamphetamine (DOB), a phenylisopropylamine (substituted amphetamine) derived solely via synthetic schemes (Shulgin and Shulgin, 1991). Various analogues of these compounds have been synthesized and tested for bioactivity (Nichols et al., 2015; Shulgin and Shulgin, 1991, 1997; Shulgin et al., 2011), but for the focus of this paper, we confine classification of CH to those drugs that have been characterized extensively in both nonhuman animals and in humans (Bogenschutz and Johnson, 2016; Halberstadt, 2015). For example, we do not classify novel N-benzyl substituted phenethylamine hallucinogens as CH, because their receptor and behavioral pharmacology are not fully characterized or diverge from CH (Nichols et al., 2008).

The fundamental, shared pharmacological property of all CH is high affinity and agonist activity at serotonin 5-HT_2_ G protein-coupled receptor (GPCR) subtypes (5-HT_2A_, 5-HT_2B_, and 5-HT_2C_), with most binding to and stimulating 5-HT_2_ receptors at low nanomolar concentrations. However, depending on the CH examined, activities at other GPCRs have been identified, particularly other 5-HT receptors, and, most notably, 5-HT_1_ receptor subtypes (Nichols, 2004; Roth et al., 2000). Many attempts have been made to uncover additional molecular targets of CH. For example, tryptamine hallucinogens display activity at the sigma-1 receptor, the serotonin transporter (SERT), and the vesicular monoamine transporter (Cozzi et al., 2009; Fontanilla et al., 2009), but high concentrations, reaching micromolar levels, are required to elicit activity, which calls into question their contribution to psychoactive effects. Also, it was reported recently that certain substituted amphetamine hallucinogens, including 2,5-dimethoxy-4-iodoamphetamine (DOI) and DOB, bind with appreciable affinity at adrenergic GPCRs (Ray, 2010). However, using traditional radioligand competition binding assays, we did not replicate the observed effects at alpha-adrenergic receptors. We obtained micromolar affinity (*K*_i_) values of (±)-DOI at each of the α-adrenergic receptor subtypes we screened (α1a, α1b, α2a, α2b, and α2c; unpublished observations; data available upon request).

Most germane to the psychoactive effects of CH is that they are blocked by 5-HT_2_ antagonists in both rodents (Fantegrossi et al., 2005) and humans (Kometer et al., 2013; Vollenweider et al., 1998). It should be noted, however, that there are exceptions wherein 5-HT_2_ antagonism is insufficient to eliminate the discriminative stimulus properties of CH (e.g., psilocybin and LSD) in rodents (Benneyworth et al., 2005; Winter et al., 2007). Clearly, pharmacodynamics and pharmacokinetics across species play an important role, and may underlie observed discrepancies (Canal et al., 2013). Nevertheless, adding to support that 5-HT_2_ receptors are the predominant mediators of CH psychoactive effects are the observations that 5-HT_2A_ knockout mice do not exhibit behaviors, such as the head-twitch response (HTR; Gonzalez-Maeso et al., 2007; Hanks and Gonzalez-Maeso, 2013), typically elicited by CH. Also, the DOI-elicited HTR is reduced in 5-HT_2C_ receptor knockout mice (Canal et al., 2010). Collectively, it can be said with confidence that 5-HT_2_ receptors predominantly mediate the psychoactive effects of CH. Here, we focus on interrogating 5-HT_2A_, 5-HT_2B_, and/or 5-HT_2C_ receptors as the elemental GPCRs that underlie their non-addictive nature.

## Results from preclinical studies and reports from experienced hallucinogen users show that classic hallucinogens have low addiction liability

One of the fascinating aspects of CH, long recognized by experienced hallucinogen users and only recently gaining traction by the broader scientific community, is that compared with most other psychoactive drugs scheduled by federal governments in the most restrictive classes (e.g., heroin in Schedule 1 and cocaine in Schedule 2), they are relatively non-addictive (Bogenschutz and Johnson, 2016). As a caveat, we understand that there can be controversy defining what are and what are not addictive substances, including defining CH as addictive or not (Nutt et al., 2010; Stone et al., 2006). Considering the DSM-5 criteria for a diagnosis of a substance-use disorder (American Psychiatric Association, 2013), we narrowly define addictive drugs as those that induce craving or an impulse to re-dose after psychoactive effects have peaked or have begun to subside. Note, this feeling and impulse is also described in the literature as “wanting” or “increased incentive salience.” Experienced drug users report that CH do not produce drug craving, which is typically elicited by other psychoactives of distinct chemical classes (e.g., methamphetamine, MDPV, nicotine, alcohol, cocaine, heroin; see erowid.org). Also, although tolerance to most CH, excluding short-acting *N,N*-dimethyltryptamine (DMT; Strassman et al., 1996), is evident with repeated, continuous exposure, withdrawal symptoms are generally non-existent once effects subside, distinguishing CH from most other drugs of abuse, including closely related serotonin-releasing empathogens such as 3,4-methylenedioxymethamphetamine (MDMA) that also have activity at dopamine transporters (Ball and Slane, 2014; Meyer, 2013).

The relatively non-addictive characteristics of CH described by human users are recapitulated in well-controlled, preclinical animal studies, which provide reliable and conclusive evidence. Such animal studies typically employ intravenous self-administration, in which an operant response by the animal (e.g., pressing a lever) is reinforced by the delivery of a dose of drug. While intramuscular (Goldberg et al., 1976), inhalation (Carroll et al., 1990a), and oral (Gomez et al., 2002) routes of administation also maintain drug-taking behavior, intravenous drug delivery is the most employed route of administration in self-administration studies, as intravenous delivery causes a rapid onset of action, facilitating learning of the association between the operant response and the psychoactive effects of the drug. Animal self-administration experiments demonstrate a very strong correlation between drugs that produce dependence in humans and those that are voluntarily consumed by laboratory animals (Brady et al., 1984; Griffiths, 1980; Griffiths et al., 1978, 1980).

One of the earliest studies on the reinforcing effects of drugs using the intravenous self-administration procedure in rhesus monkeys found that no animal initiated self-injection of mesca-line either spontaneously or after one month of programmed administration, and this was apparent at doses that produced physiological and psychoactive effects such as salivation, mydriasis, pilo-erection, and apparent apprehension (Deneau et al., 1969). The lack of mescaline self-administration stood in contrast to positive findings of self-administration of morphine, codeine, cocaine, amphetamine, pentobarbital, ethanol, and caffeine. A subsequent study with rhesus monkeys using 2,5-dimethoxy-4-methylamphetamine (DOM; Yanagita, 1986) provided similar results as the mescaline study. These findings have withstood the test of time, as the primary literature is virtually devoid of any accounts of self-administration of CH, suggesting that there are very limited conditions under which laboratory animals voluntarily consume CH. In one example, extreme environmental conditions were required to elicit self-administration of DMT in monkeys (Siegel and Jarvik, 1980). In this study, under baseline conditions, monkeys sampled DMT but did not engage in significant or persistent self-administration. However, following several days of sensory deprivation (i.e., the absense of light and sound), two of the three monkeys consistently self-administered DMT for up to 20 days (which was the a priori imposed endpoint of the experiment). These observations were conceptualized as the DMT engendering an internal or mental perceptual window with concomitant positive reinforcing effects in the context of sensory deprivation. In a second example, various hallucinogens were substituted in monkeys that were maintained on a baseline of (±)-MDMA self-administration. Although none of the subjects consistently self-administered any of the hallucinogens tested, all of the subjects transiently and sporadically self-administered psilocybin, mescaline, and DMT at response rates that were comparable to MDMA and up to the maximum number of infusions that were available (Fantegrossi et al., 2004a). These effects, however, were transient and sporadic, and clearly unlike those seen across a broad range of other psychoactive drugs. In our studies, we observed very weak reinforcing effects of psilocybin in rhesus macaque monkeys. Once responding during saline administration was procedurally manipulated to an extradorinarily low level (approximately 0.01 responses per second), some positive reinforcing effects were apparent with psilocybin (unpublished observations; data available upon request), but yet again, these effects massively pale in comparison to other drugs of abuse (Murnane et al., 2013b). Based on these studies, it is clear that conditions have not yet been found, despite decades of efforts, wherein CH are readily self-administered by laboratory animals. Given the very strong correlation between drugs that produce dependence in humans and those that are voluntarily consumed by laboratory animals (Brady et al., 1984; Griffiths, 1980; Griffiths et al., 1978, 1980), this is among the strongest evidence for the non-addictive nature of CH.

Despite the relative absence of addictive properties, observed in well-controlled preclinical studies and reported by human users, CH can dose-dependently elevate mood and produce a strong sense of well-being, depending on mental set and environmental setting (Studerus et al., 2012). In fact, many people who have taken CH report that their experiences were of the most influential and positive of their lives (Griffiths et al., 2011). These positive psychological effects of CH, nonetheless, do not qualify them as without risk, especially when taken without precaution, careful planning, and supervision. CH produce profound alterations in cognition and sensory perception and can produce emotional lability, fear, anxiety, and/or panic, among other adverse psychological and physiological effects. Nonetheless, if CH can elevate mood and produce a sense of well-being, why are they non-addictive?

## Why are classic hallucinogens non-addictive? Focus on serotonin

There are several biological possibilities to explore why CH are relatively non-addictive. For example, the long durations of action of many CH and their capacity to induce 5-HT_2A_ receptor desensitizaton (Leysen et al., 1989), which causes rapid, prolonged, and profound tolerance to their psychoactive effects (i.e., tachyphalaxis), may be factors. In support of these possibilities, it is well known that long pharmacokinetic profiles, including long durations of action, can minimize abuse liability (Abreu et al., 2001; Fantegrossi et al., 2004b; Marsch et al., 2001; Nelson et al., 2006; Volkow et al., 2000; Woolverton and Wang, 2004). The psychedelic effects of LSD, which persist for approximately 8–12 hours, are greatly attenuated with repeated dosing; a few days of abstinence are required before subjective effects return to pre-exposure levels (Belleville et al., 1956). Thus, spacing intake of LSD is necessary, which prevents the binge-like patterns of drug consumption often seen with highly addictive substances (Aarde et al., 2015; Roberts et al., 2007). However, effects of DMT are inconsistent with this explanation. The hallucinogenic effects of DMT commence within two minutes of intravenous administration, and subside by 30 minutes (Strassman and Qualls, 1994). Moreover, the psychedelic effects of DMT persist with repeated administration in a single drug-taking session (Strassman, 1996). This may generalize across some tryptamines, as a recent study shows profound tolerance in mice to two phenethylamines (DOI and 2C-T-7), but a lack of tolerance to two tryptamines (DPT and DIPT; Smith et al., 2014). Despite their differerent durations of action and capacities to induce tolerance, none of the CH mentioned above are known to produce drug craving in human users. Comparatively, re-intoxication from addictive psychoactives can be achieved quickly after their effects subside, despite long durations of action (e.g., MDMA) combined with target densitization and/or internalization and tolerance. For example, cannabinoid CB_1_ receptor downregulation is evident with prolonged cannabis use (Ceccarini et al., 2015), but the subjective high from cannabis persists, even with tolerance and daily use. To maintain scope, this review focuses on serotonin receptor targets of CH that likely underlie their non-addictive properties.

The reinforcing and addictive effects of psychoactive drugs are primarily attributed to drug-induced changes in central dopamine function. Pointedly, the transition to addiction is mediated by increases in firing of ventral tegmental area (VTA) dopamine neurons projecting to the nucleus accumbens (NAc), which increases dopamine release in the NAc (Cline et al., 1992; Kuhar et al., 1999; Pascoli et al., 2015; Ritz et al., 1989; Robison and Nestler, 2011); all addictive drugs increase dopamine release in the NAc. Serotonin, on the other hand, plays an important modulatory role in the behavioral effects of many psychoactive drugs. Serotonin neurons originate in the raphe nuclei in the brainstem, and send strong projections to the VTA, prefrontal cortex (PFC), amygdala, hippocampus, dorsal striatum, and NAc (Halliday and Tork, 1989). Serotonergic projections innervate cell bodies and terminals of dopamine neurons (Beart and McDonald, 1982; Geyer et al., 1976; Nedergaard et al., 1988; Parent et al., 1981), often making direct synaptic contact (Herve et al., 1987; Nedergaard et al., 1988), and serotonin provides tonic and phasic control of dopaminergic systems within the limbic pathway (Alex and Pehek, 2007).

Converging results from many studies demonstrate that enhancing central serotonin release attenuates addictive behaviors (Muller and Homberg, 2015). For example, selective stimulation of serotonergic dorsal raphe nucleus afferents to the NAc, using designer receptors exclusively activated by designer drugs (DREADDs), abolishes cocaine-elicited conditioned place preference (You et al., 2016). Furthermore, there is a negative relationship between the potencies of several cocaine- and amphetamine-like analogs in self-administration studies and their binding potencies as SERT inhibitors (Ritz and Kuhar, 1989; Ritz et al., 1989). For example, rhesus monkeys self-administer more infusions of PAL-353, which has high selectivity for releasing dopamine versus serotonin, than PAL-313, which non-selectively releases dopamine and serotonin (Wee et al., 2005). Similarly, rhesus monkeys do not self-administer PAL-287, which is relatively nonselective at releasing dopamine and serotonin, across a range of doses (Rothman et al., 2005). Adding to the evidence, systemic administration of selective SERT inhibitors, which selectively increase extracellular levels of serotonin, decrease cocaine self-administration in rodents (Carroll et al., 1990b; Richardson and Roberts, 1991) and nonhuman primates (Kleven and Woolverton, 1993). In nonhuman primates, SERT inhibitors, such as citalopram, fluoxetine, and alaproclate, attenuate the behavioral-stimulant effects of cocaine, cocaine self-administration, cocaine-induced increases in extracellular dopamine, and cocaine-induced activation of the PFC (Czoty et al., 2002; Howell and Byrd, 1995; Howell et al., 2002; Spealman, 1993). Additional studies show that SERT inhibitors attenuate drug-induced increases in dopamine levels in rodents and nonhuman primates (Czoty et al., 2002; Di Matteo et al., 2008) and cue-induced reinstatement of extinguished cocaine-maintained lever-pressing behavior in rats (Baker et al., 2001; Burmeister et al., 2003). Furthermore, an early study shows that serotonin infused directly into the NAc attenuates locomotor activity stimulated by direct infusions of dopamine into the NAc (Jones et al., 1981). These studies demonstrate convincingly that serotonin opposes the effects of dopamine and attenuates the abuse-related and addictive effects of psychoactive drugs (for reviews, see Howell and Murnane, 2008; Murnane and Howell, 2011).

While it is well documented that serotonin attenuates the reinforcing effects of a variety of psychoactive drugs through suppression of dopamine neurotransmission, it is important to consider that there are 16 distinct serotonin receptors (not including splice variants or RNA-edited 5-HT_2C_ receptors) that are grouped into seven families: 5-HT_1_–5-HT_7_ receptors (Boess and Martin, 1994; Green, 2006; Hoyer et al., 1994, 2002). Unique serotonin receptors can facilitate, inhibit, or have no effect on dopamine neurotransmission and on the reinforcing properties of a variety of psychoactive drugs. With particular relevance to the anti-addictive properties of CH, there are a number of reported observations of opposing effects between the 5-HT_2C_ and 5-HT_2A_ receptor subtypes, with 5-HT_2A_ receptors facilitating and 5-HT_2C_ receptors suppressing dopamine neurotransmission (Bubar and Cunningham, 2008; De Deurwaerdere and Spampinato, 1999; Filip et al., 2004, 2006; Fletcher et al., 2002; McMahon and Cunningham, 2001; McMahon et al., 2001). The divergent effects of 5-HT_2A_ and 5-HT_2C_ on dopamine neurotransmission are at first consideration peculiar, since 5-HT_2_ receptor subtypes couple to the same G-proteins (e.g., Gα_q/11/12/13_) and activate the same intra-cellular signaling pathways (e.g., phospholipase C [PLC], mitogen-activated protein kinase, β-arrestin), with a few notable exceptions (Berg et al., 1994, 2001; Chagraoui et al., 2016; Knauer et al., 2009; Sanders-Bush et al., 2003). However, the differences observed may also be due to differences in the relative expression of 5-HT_2A_ and 5-HT_2C_ receptors across cell types and microcircuits that control unique aspects of neural function, a point discussed in greater detail in the 5-HT_2C_ sections below. Nonetheless, the differences between 5-HT_2A_ and 5-HT_2C_ modulation of dopamine neurotransmission provide a clear hypothesis for why CH, which do not provide a large spread between 5-HT_2C_ and 5-HT_2A_ receptor activation, do not induce craving. We argue that activation of 5-HT_2C_ receptors tempers the addictive liability of CH.

## Considerations of 5-HT_2A_ receptor activation to addiction liability

There is a substantial body of evidence that the 5-HT_2A_ receptor facilitates dopamine neurotransmission. For example, systemic administration of DOI increases the firing rates of dopamine neurons in the VTA and induces dopamine release in the PFC, effects that are attenuated by selective antagonism of the 5-HT_2A_ receptor (Bortolozzi et al., 2005; Pehek et al., 2006) or genetic deletion of the 5-HT_2A_ receptor (Di Matteo et al., 2000; Huang et al., 2011). The preponderance of evidence for 5-HT_2A_ receptor facilitation of dopamine neurotransmission emanates from studies of the mesocortical system because of the widespread interest in serotonin modulation of this system in schizophrenia. Nevertheless, some studies examined 5-HT_2A_ receptor modulation of dopamine neurotransmission within the NAc, which is a key region in the mesolimbic dopamine pathway critical for addiction (Haber and Knutson, 2010; Russo and Nestler, 2013; Sesack and Grace, 2010). In this regard, direct administration of DOI into the posterior NAc significantly increases dopamine levels locally within the NAc, effects that are blocked by 5-HT_2A_ antagonists (Bowers et al., 2000; Yan, 2000). However, we recently showed that systemic administration of DOI in rhesus monkeys engenders only meager (∼10% increase above baseline) elevations in dopamine levels in the NAc (unpublished observations; data available upon request), and similarly, previous studies in rats show that systemic DOI does not affect dopamine release in the NAc (Di Matteo et al., 2000). This suggests that DOI acting on additional neural systems or microcircuits suppresses the increase in dopamine neurotransmission in the NAc.

That 5-HT_2A_ receptors may contribute to drug seeking is further supported by studies employing neuroimaging, behavioral pharmacology, and genetic tools. A recent primate neuroimaging study shows that several months of cocaine self-administration increases the availability of 5-HT_2A_ receptors in the PFC, suggesting that increased 5-HT_2A_ receptor availability facilitates drug-taking behavior (Sawyer et al., 2012). This is supported by observations that 5-HT_2A_ knockout mice self-administer MDMA to a lesser degree than wild-type mice, and that the selective 5-HT_2A_ inverse agonist, eplivanserin, blocks cue-induced reinstatement of MDMA seeking (Orejarena et al., 2011). Other studies report that 5-HT_2A_ receptor antagonists attenuate the stimulatory effects of cocaine, amphetamine, MDMA, and methamphetamine on dopamine neurotransmission as well as their locomotor stimulant and interoceptive effects (Auclair et al., 2004; Broderick et al., 2004). Intra-VTA microinjections of 5-HT_2A_ receptor antagonists attenuate cocaine- and amphetamine-induced increases in motor activity in mice (Auclair et al., 2004) and rats (McMahon et al., 2001), and we showed recently that selective 5-HT_2A_ receptor antagonism attenuates the dopamine releasing and behavioral effects of amphetamine in primates (Murnane et al., 2013a). Systemic administration of 5-HT_2A_ receptor antagonists (including SR 46349B, M100907, and MDL 11,939), in contrast to the effects of agonists, do not alter firing rates of dopamine neurons in the VTA or dopamine release in the NAc or PFC of rodents (Bonaccorso et al., 2002; Di Giovanni et al., 1999; Gobert et al., 2000; Pehek et al., 2006; Porras et al., 2002). Therefore, although the 5-HT_2A_ receptor does not appear to exert a tonic influence on dopamine neuronal firing or release, agonist stimulation of this receptor enhances dopaminergic activity (Bubar and Cunningham, 2008). Though, net effects may depend on specific cell types and circuits examined.

The use of reinstatement procedures provides an important complement to drug self-administration, as it is the most widely accepted model of drug relapse. As drug relapse is critically influenced by craving and withdrawal, reinstatement experiments have particular relevance for understanding the non-addictive nature of CH. Such studies show that 5-HT_2A_ receptor antagonism prevents reinstatement of drug-seeking behavior. For example, the 5-HT_2_ receptor antagonist ketanserin attenuates cue-induced reinstatement of extinguished cocaine self-administration in rats (Burmeister et al., 2004), and the selective 5-HT_2A_ receptor antagonist M100907 attenuates both drug- and cue-induced reinstatement of extinguished cocaine self-administration in rats (Fletcher et al., 2002; Nic Dhonnchadha et al., 2009). Consistent with these findings, we have shown that 5-HT_2A_ receptor antagonism attenuates cue- as well as drug-induced reinstatement of extinguished behavior that was previously maintained by cocaine in rhesus monkeys (Murnane et al., 2013b).

These studies suggest that selective activation of 5-HT_2A_ receptors may induce craving and/or relapse. Nevertheless, it will be critical to assess empirically the addictive or anti-addictive potential of highly selective 5-HT_2A_ agonists. A recent report shows that an analogue from the phenethylamine class of CH, (4-bromo-3,6-dimethoxybenzocyclobuten-1-yl)methylamine (TCB-2) that has high 5-HT_2A_ agonist potency (McLean et al., 2006) reduces intracranial self-stimulation (ICSS), but does not attenuate the potentiating effects of cocaine on ICSS (Katsidoni et al., 2011). Its activity at 5-HT_2B_ and 5-HT_2C_ receptors, however, has not been reported to our knowledge. Finally, CH may be biased agonists (Gonzalez-Maeso and Sealfon, 2009; McLean et al., 2006; Wacker et al., 2013) that stabilize 5-HT_2A_ receptor active conformations that block craving. Certainly, additional studies are warranted.

## Considerations of 5-HT_2B_ receptor activation to addiction liability

Owing to their low expression in the central nervous system (Bonhaus et al., 1995; Duxon et al., 1997; Kursar et al., 1994; Loric et al., 1992), much less attention has been given to the potential role of 5-HT_2B_ receptors to the effects of psychoactive substances. Moreover, as prolonged activation of 5-HT_2B_ receptors on heart valve leaflets is linked to valvular heart disease (Hutcheson et al., 2012; Roth, 2007; Rothman and Baumann, 2009), an impetus to develop highly-selective 5-HT_2B_ agonists has been lacking. Indeed, there is not a highly selective 5-HT_2B_ agonist probe that is a widely accepted tool of the scientific community. Thus, there are somewhat limited data regarding the contribution of 5-HT_2B_ receptor activation to psychoactive substances. Much of what is known, however, emanated predominantly from the Maroteaux laboratory. Converging data from 5-HT_2B_ receptor knockout mice and from use of 5-HT_2B_ ligands show that central 5-HT_2B_ receptors contribute to the psychomotor effects of MDMA (Doly et al., 2008), the anorexient effects of dexfenfluramine (Banas et al., 2011), and the antidepressant effects of selective serotonin reuptake inhibitors (Diaz et al., 2012; Hertz et al., 2015). Another recent study shows that LY266097, a selective 5-HT_2B_ antagonist, reduces basal and amphetamine-stimulated dopamine release in the NAc, and also decreases amphetamine-elicited hyperlocomotion (Auclair et al., 2010), although others report that selective 5-HT_2B_ antagonists, including SB 204741, do not affect cocaine-elicited hyperlocomotion or the discriminative stimulus effects of cocaine (Filip et al., 2004, 2006). In addition to the different neuropharmaco-logical effects of amphetamine versus cocaine, the potential discrepancies in these studies could relate to the use of different 5-HT_2B_ antagonist ligands, and consequently target selectivity or off-target liability.

Finally, Roth's group reported that the designer empathogen 1-(benzofuran-6-yl)propan-2-amine (6-APB), an MDMA analogue, binds with >100-fold selectivity to the 5-HT_2B_ receptor compared to 5-HT_2A_ or 5-HT_2C_ receptors, and furthermore, 6-APB is a 5-HT_2B_ receptor agonist (Iversen et al., 2013). Interestingly, 6-APB has higher affinity at 5-HT_2B_ than any other target tested, including the dopamine transporter, a primary target of addictive psychostimulants (e.g., cocaine and methamphetamine). Anecdotal reports note that 6-APB produces subjective effects similar to MDMA, but according to some, it does not produce drug craving and has a low propensity to cause physical dependence (see bluelight.org and erowid.org). However, controlled tests of its addictive potential have not been reported. Additional studies assessing the contribution of 5-HT_2B_ receptor activation to the low addiction liability of CH are warranted.

## Considerations of 5-HT_2C_ receptor activation to addiction liability: Behavioral studies

Numerous lines of evidence from multiple laboratories show that 5-HT_2C_ receptor activation attenuates self-administration of addictive substances and also attenuates ICSS of the brain's primary reward circuitry, and there are a number of excellent recent reviews on the topic of 5-HT_2C_ receptor agonists for addiction and mechanisms underlying their effects (De Deurwaerdere et al., 2013; Devroye et al., 2013; Di Giovanni et al., 2006; Higgins and Fletcher, 2015; Howell and Cunningham, 2015; Muller and Homberg, 2015). Selective agonists of the 5-HT_2C_ receptor have generally been found to recapitulate the attenuating effects of indirect serotonin agonists on behavioral models of addiction, suggesting 5-HT_2C_ receptor activation is a key molecular mechanism by which serotonin exerts its anti-addictive effects. For example, the rate-suppressing effects of both the indirect serotonin receptor agonist fenfluramine and the 5-HT_2C_ receptor agonist Ro 60-0175 in an ICSS procedure are blocked by pretreatment with the 5-HT_2C_ receptor selective antagonist SB 242084 (Bauer et al., 2015). Likewise, Ro 60-0175 blocks cocaine-seeking behavior in rats, an effect completely reversed by SB 242084 (Burbassi and Cervo, 2008). Acute VTA or systemic administration of a variety of relatively selective 5-HT_2C_ receptor agonists in rodents suppress the locomotor stimulant effects of cocaine (Craige and Unterwald, 2013; Filip et al., 2004; Fletcher et al., 2004; Grottick et al., 2000), the discriminative stimulus effects of cocaine (Callahan and Cunningham, 1995; Cunningham et al., 2011; Fletcher et al., 2008; Frankel and Cunningham, 2004), cocaine self-administration (Cunningham et al., 2011; Fletcher et al., 2008; Grottick et al., 2000), and reinstatement of cocaine seeking induced by exposure to cocaine and cocaine-associated cues (Burbassi and Cervo, 2008; Cunningham et al., 2011; Fletcher et al., 2008; Grottick et al., 2000; Neisewander and Acosta, 2007). These findings have been extended to other classes of psychoactive drugs, including alcohol, cannabis, and nicotine (Higgins and Fletcher, 2015; Ji et al., 2006; Marcinkiewcz, 2015; Quarta et al., 2007; Zaniewska et al., 2007).

In primates, recent studies show that Ro 60-0175 reduces psychostimulant effects of cocaine, cocaine self-administration, and cocaine-induced reinstatement (Manvich et al., 2012b; Ruedi-Bettschen et al., 2015). These effects do not appear to result from general suppression of operant responding, as the same dose of Ro 60-0175 has no effect on operant responding maintained by negative reinforcement (Manvich et al., 2012b). Similarly, the highly-selective 5-HT_2C_ receptor agonist lorcaserin attenuates the discriminative stimulus effects of cocaine and suppresses cocaine self-administration following acute or 14-day treatment in rhesus monkeys (Collins et al., 2016). Clinical trials assessing lorcaserin's ability to reduce cocaine self-administration are now underway (e.g., ClinicalTrials.gov Identifier: NCT02537873).

The hypothesis that 5-HT_2C_ receptors inhibit a variety of abuse-related effects of drugs is substantiated by the reliable finding that antagonists facilitate such effects. In rodents, systemic administration of 5-HT_2C_ receptor antagonists enhance the locomotor stimulant effects of cocaine (Fletcher et al., 2002, 2006), the discriminative stimulus effects of cocaine (Filip et al., 2006), cocaine self-administration (Fletcher et al., 2002), and cocaine-induced reinstatement (Fletcher et al., 2002). Consistent findings are reported in primates, as SB 242084 increases rates of responding maintained under a fixed-interval schedule of stimulus termination, increases cocaine-primed reinstatement of cocaine self-administration, and, critically, maintains self-administration when substituted for cocaine (Manvich et al., 2012a). Other studies also suggest that 5-HT_2C_ antagonists themselves may be addictive substances (Di Giovanni et al., 1999; Di Matteo et al., 1999). Finally, 5-HT_2C_ receptor knockout mice exhibit increased sensitivity to the effects of cocaine (Rocha et al., 2002). Collectively, the literature is replete with evidence that activation of 5-HT_2C_ receptors inhibits addictive effects of a variety of drugs. As CH activate 5-HT_2C_ receptors, we surmise this property is an essential facet that renders CH non-addictive.

## Considerations of 5-HT_2C_ receptor activation to addiction liability: Neurochemistry studies

The most supported mechanism for the anti-addiction effects of 5-HT_2C_ activation is inhibition of mesolimbic dopamine neurons, which decreases psychostimulant-elicited dopamine release in the NAc (Alex and Pehek, 2007; Bubar et al., 2011; Collins et al., 2016; Di Giovanni et al., 2000; Di Matteo et al., 2000; Fletcher et al., 2008; Harvey-Lewis et al., 2016; Howell and Cunningham, 2015; Manvich et al., 2012b; Navailles et al., 2008; Rocha et al., 2002). Evidence suggests these effects are caused by 5-HT_2C_ receptor-mediated activation of GABA neurons in the VTA, which directly inhibit VTA dopamine neuron activity (Bubar and Cunningham, 2007; Bubar et al., 2011; Di Giovanni et al., 2001; Howell and Cunningham, 2015). Conversely, inactivation of 5-HT_2C_ receptors by antagonists or inverse agonists increases firing rates of VTA dopamine neurons, increasing dopamine levels in the NAc (Di Giovanni et al., 1999; Di Matteo et al., 2004). Identifying the specific neural circuitry examined in such studies, however, is important, as these observations ostensibly conflict with the conclusions from a new report about 5-HT_2C_ receptor modulation of dopamine neurotransmission (Xu et al., 2016). Specifically, using slice electrophysiology, the authors report that 5-HT_2C_ receptors expressed on dopamine neurons stimulate a significant number of them. Collectively, the data suggest that the emergent effects of 5-HT_2C_ activation on dopamine neurotransmission depend on the relative activation of 5-HT_2C_ receptors on different cell types; for example, GABA neurons innervating dopamine neurons (resulting in dopamine neuron inhibition) relative to 5-HT_2C_ receptors expressed directly on dopamine neurons (resulting in dopamine neuron activation). In support of this, in mice that express 5-HT_2C_ receptors only on dopamine VTA neurons, 5-HT_2C_ activation enhances dopamine neuron activity to a greater degree than in wild-type mice, suggesting that 5-HT_2C_ receptors on other cell types have an inhibitory effect on dopamine neurotransmission (Xu et al., 2016).

**Figure 1:**
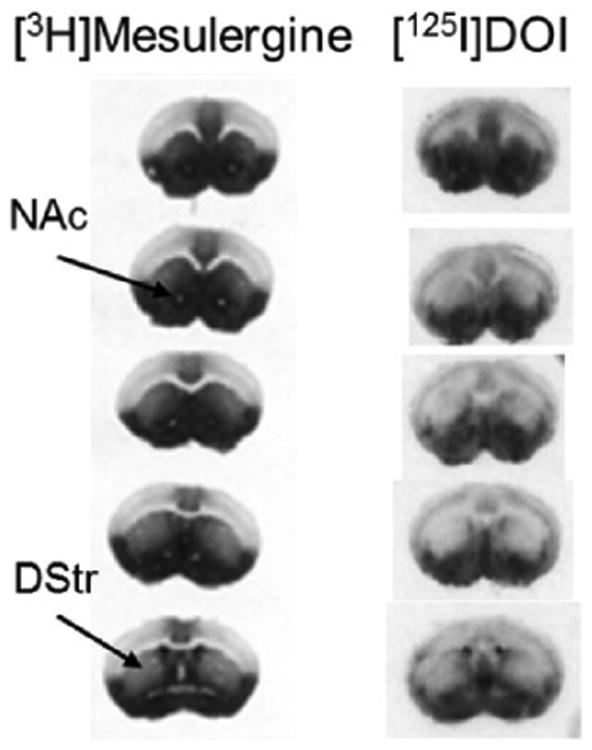
Autoradiographs of brain 5-HT_2C_ receptors from mice that overexpress 5-HT_2C_ (5-HT_2C-VGV_) allowing clear observations of 5-HT_2C_ receptor distribution in the brain. Notably, 5-HT_2C_ is densely expressed in the nucleus accumbens (NAc), but not in the dorsal striatum (DStr); the darker the shade, the higher the receptor binding site density. [^3^H]Mesulergine (3 nM for eight weeks) or [^125^I]DOI (0.14 nM for 48 hours) was used in the presence of spiperone (100 nM) to label 5-HT_2C_ receptors. These sections were part of a set collected by Dr. Canal (Olaghere da Silva et al., 2010). All sections labeled with [^3^H] Mesulergine or with [^125^I]DOI are from the same brain. Pictures from the latter were cropped and pasted to align them vertically.

Other findings corroborate the view that 5-HT_2C_ receptor activation impacts reward circuitry via multiple mechanisms. Recent studies point to direct modulatory effects of 5-HT_2C_ receptor activation on dopamine signaling in the NAc as contributors to their anti-addiction properties, and these effects may have as much relevance for addiction treatment as the effects of 5-HT_2C_ receptors in the VTA. For example, 5-HT_2C_ receptors expressed in the NAc inhibit postsynaptic dopamine signaling by inhibiting phosphorylation of DARPP-32, independent of dopamine release (Cathala et al., 2015; Devroye et al., 2015). 5-HT_2C_ receptor knockout mice also provide evidence for 5-HT_2C_ negative modulation of dopamine neurotransmission in reward circuitry. 5-HT_2C_ knockout mice are more sensitive to the psychostimulant effects of amphetamine, cocaine, and GBR 12909 (another dopamine reuptake blocker) and also exhibit increased cocaine-stimulated release of dopamine in their NAc (Abdallah et al., 2009; Rocha et al., 2002). Interestingly, the enhanced effects of cocaine on dopamine release in the NAc in 5-HT_2C_ knockout mice are not observed in the dorsal striatum (Rocha et al., 2002). Similarly, amphetamine's effects on dopamine release in the dorsal striatum are not potentiated in 5-HT_2C_ knockout mice (Abdallah et al., 2009). This neural system-specific effect is recapitulated using 5-HT_2C_-selective ligands. For example, 5-HT_2C_ agonists administered systemically attenuate cocaine-stimulated dopamine release in the NAc, but not the dorsal striatum in wild-type mice (Di Giovanni et al., 2000) or in nonhuman primates (Manvich et al., 2012b). Also, systemic treatment with 5-HT_2C_ agonists alone reduces dopamine in the NAc, but not the dorsal striatum (Di Giovanni et al., 2000; Marquis et al., 2007), and the converse effects are observed after treatment with 5-HT_2C_ antagonists or inverse agonists; that is, they increase dopamine release in the NAc, but again, they have minimal effects on dopamine release in the dorsal striatum (Di Matteo et al., 1999). These data are confluent with the distribution of 5-HT_2C_ receptors across neural systems, as 5-HT_2C_ receptors are densely expressed in the NAc but not the dorsal striatum (Figure 1). These observations suggest that post-synaptic 5-HT_2C_ receptors in the NAc may contribute strongly to the effects of selective 5-HT_2C_ agonists, administered systemically, on dopamine release in reward circuitry, a hypothesis we build in the following sections. Overall, the data provide a mechanistic rationale for the efficacy of CH, as 5-HT_2C_ agonists, to temper the rewarding effects of addictive drugs. As a recent optogenetic study showed exquisitely that dopamine cell firing in the medial forebrain bundle, including the VTA—NAc tract, is sufficient to cause a transition to addiction (Pascoli et al., 2015), it is clear that 5-HT_2C_ agonists have promise as tools in the treatment of addiction.

## The NAc is a key site for 5-HT_2C_ receptor modulation of reward circuitry

As noted above, much of the circuitry-related data supporting 5-HT_2C_ receptor modulation of psychostimulant effects is focused on 5-HT_2C_ receptors expressed on GABA neurons of the VTA. We do not discount these findings. However, important rationale for our focus on 5-HT_2C_ receptors in the NAc are the observations that infusions of the selective 5-HT_2C_ agonist, WAY 161503, into the NAc, but not the VTA, decrease the reward-facilitating effects of cocaine (Katsidoni et al., 2011). Moreover, infusions of the selective 5-HT_2C_ antagonist, SB 242084, into the VTA does not affect cocaine-induced dopamine release in the NAc, but when infused into the NAc, SB 242084 potentiates cocaine-induced dopamine release in the NAc (Navailles et al., 2008). Also, the 5-HT_2C_ inverse agonist, SB 206553 increases basal dopamine release in the NAc when it is infused there, but not when it is infused in the VTA, suggesting 5-HT_2C_ constitutive activity in the NAc, but not VTA, modulates tonic NAc dopamine release (De Deurwaerdere et al., 2013; Navailles et al., 2006). In summary, these data suggest that activation of NAc 5-HT_2C_ receptors negatively modulates the effects of cocaine.

Raphe nuclei send strong 5-HT projections to the NAc (Van Bockstaele and Pickel, 1993), and 5-HT_2C_ receptors are predominantly post-synaptic and excitatory (Austgen et al., 2012; Invernizzi et al., 2007), suggesting they reside on GABAergic MSN, which account for ∼90% of NAc neurons (Meredith, 1999). Indeed, immunochemistry and neurophysiology studies show that 5-HT_2C_ receptors are expressed on MSN of the NAc (Clemett et al., 2000; Graves et al., 2015; Santana and Artigas, 2016). Furthermore, autoradiography data from mice that over-express 5-HT_2C_ receptors (while maintaining the distribution pattern of 5-HT_2C_ receptors across neural systems that is observed in wild-type mice; Olaghere da Silva et al., 2010) show clearly that 5-HT_2C_ receptors are densely expressed in the NAc, but not the dorsal striatum (see Figure 1; which receives dopaminergic projections from substantia nigra). This is particularly intriguing when considered in the context of observations of the effects of cocaine on dopamine release in the NAc compared with the dorsal striatum. As stated earlier, 5-HT_2C_ knockout mice, relative to WT mice, show an increase in cocaine-stimulated dopamine release in the NAc, but this effect is not observed in the dorsal striatum (Rocha et al., 2002). Furthermore, 5-HT_2C_ agonists administered peripherally suppress cocaine-stimulated dopamine release in the NAc, but not the dorsal striatum (Manvich et al., 2012b). Thus, we consider, like others (Navailles et al., 2006, 2008), that 5-HT_2C_ receptors expressed in NAc are contributing significantly to the anti-addiction effects of 5-HT_2C_ agonists, with special relevance to cocaine addiction.

Several studies show that the shell region of the NAc is associated more closely than the core with the appetitive or reinforcing effects of addictive substances (Bari and Pierce, 2005; Di Chiara et al., 2004; Ikemoto, 2010; Rodd-Henricks et al., 2002; Sellings and Clarke, 2003). For example, non-contingent and self-administered cocaine preferentially increases dopamine in the NAc shell (Lecca et al., 2004, 2007; Pontieri et al., 1995), and a recent predictive model shows that cocaine treatment enhances the phasic signaling of dopamine neurons projecting to the NAc shell, but not to the core (Dreyer et al., 2016). Also, others report, more generally, that the NAc shell codes reward value (Saddoris et al., 2013). Furthermore, MSN of the NAc shell, specifically D1-containing MSN, send direct projections to the VTA, generating an inhibitory feedback circuit (Bocklisch et al., 2013; Sesack and Grace, 2010); this circuit is necessary for the integration of our 5-HT_2C_ mechanistic hypothesis, and 5-HT_2C_ regulation of NAc-VTA circuitry has been speculated (Filip and Cunningham, 2002). Interestingly, 5-HT projections to the NAc segregate in the core and shell regions; 5-HT terminals in the NAc shell are larger in diameter, contain more, large dense core vesicles, and form more symmetric contacts with dendrites (Brown and Molliver, 2000; Van Bockstaele and Pickel, 1993). Moreover, systemic administration of 5-HT_2C_ selective agonists decrease, and inverse agonists increase, dopamine release in the NAc shell (De Deurwaerdere et al., 2004; Di Matteo et al., 2000; Gobert et al., 2000). Finally, immunohistochemistry results show that 5-HT_2C_ receptors are expressed at higher densities in the NAc shell, relative to the core (Clemett et al., 2000). Thus, we speculate that 5-HT_2C_ receptors are expressed on D1-containing MSN of the NAc shell, and when activated, they enhance their activity, occluding effects of D1 activity, and thus increasing GABA release in the VTA. This is supported by the observations that 5-HT_2C_ receptor knockout mice show enhanced behavioral responses to the D1 receptor agonist SKF 81297 (Abdallah et al., 2009).

## 5-HT_2C_ modulation of intrinsic plasticity via inhibition of NAc Kv1.x channels: A novel hypothesized mechanism for psychostimulant addiction treatment

**Figure 2:**
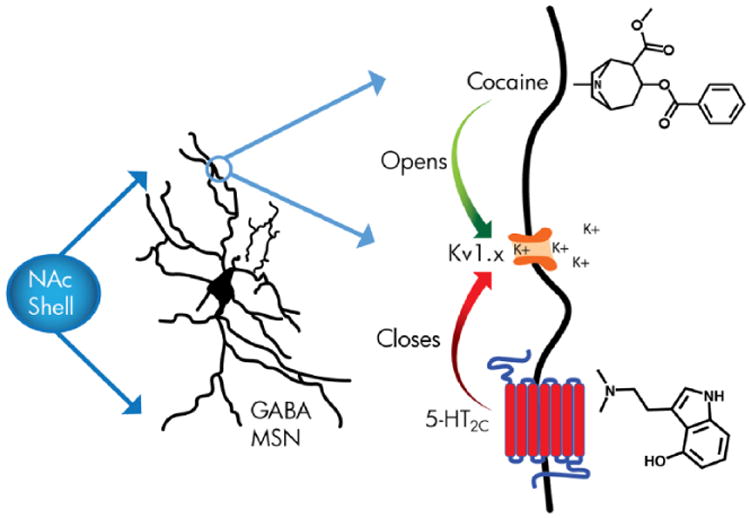
Proposed 5-HT2C anti-cocaine addiction mechanism involving modulation of Kv1.x channels on GABAergic medium spiny neurons (MSN) of the NAc shell.

Based on a synthesis of what is known in the literature regarding 5-HT_2C_ receptor function, neural circuitry, and neurochemistry underlying addiction, we hypothesize that activation of 5-HT_2C_ receptors, specifically on GABAergic MSN in the NAc shell (Graves et al., 2015), inhibits Kv1.x channels, including Kv1.1, Kv1.2, and Kv1.3, leading to increased intrinsic activity (non-synaptic increases in neuronal firing capacity). Taken together with several reports that show NAc intrinsic activity is decreased by psychostimulant exposure (Coffey et al., 2015; Graves et al., 2015; Henry and White, 1995; Hu, 2007; Hu et al., 2004; Zhang et al., 2002), and germane to our hypothesis that Kv1.x channel conductance is enhanced by exposure to psychostimulants (Hu et al., 2004; Kourrich and Thomas, 2009; Kourrich et al., 2013, 2015), it is inferred that 5-HT_2C_ receptor activation may directly counteract effects of psychostimulants on intrinsic plasticity. Accordingly, the increase in NAc shell MSN cellular activity by 5-HT_2C_ receptor activation would directly counteract the decrease in NAc shell MSN activity caused by psychostimulants (Figure 2).

Potassium Kv1.x channels (Chandy and Gutman, 1993), Kv1.1–Kv1.8, are voltage-gated channels that regulate the intrinsic activity of neurons. Kv1.x channel conductance is critical for the generation and modulation of action potentials, regulating neurotransmitter release and neural circuit excitability. Kv1.x channels open upon membrane depolarization, permitting the flow of potassium ions from within the cell, leading to restoration of the resting membrane potential. In addition, activation of Kv1.x inhibits cell-firing frequency and delays the onset of action potentials (Lioudyno et al., 2013). Conversely, Kv1.x channel blockers prevent the flow of potassium ions, causing spontaneous depolarization and increasing, for example, action potential frequency and neurotransmitter release (Ramirez-Navarro et al., 2011; Simeone et al., 2013; Tibbs et al., 1996). There are several excellent reviews on Kv channel localization in the brain, their trafficking, structure, and function, and involvement in brain disease pathophysiology (D'Adamo et al., 2013; Heusser and Schwappach, 2005; Johnston et al., 2010; Robbins and Tempel, 2012; Robertson, 1997; Shah and Aizenman, 2014; Wang et al., 1994).

Several studies, using both ex vivo and in vitro cell systems show clearly that 5-HT_2C_ activation suppresses Kv1.x channels. First, 5-HT_2C_ receptors are expressed in neural systems where Kv1.x channels are also expressed (D'Adamo et al., 2013). For example, the choroid plexuses, where 5-HT_2C_ receptor expression is dense (Hartig et al., 1990; Lopez-Gimenez et al., 2001; Marazziti et al., 1999), also robustly express Kv1.1 and Kv 1.3 channels (Speake et al., 2004). Application of 5-HT to choroid plexus cells abolishes potassium currents, an effect reversed by addition of the 5-HT_2C_ antagonist, mesulergine (Speake et al., 2004). Similarly, in heterologous oocyte systems expressing 5-HT_2C_ receptors and Kv1.1, Kv1.2, or Kv1.3 channels, application of 5-HT eliminates potassium generated currents from each channel (Aiyar et al., 1993; Imbrici et al., 2000). The effect of 5-HT_2C_ may be relegated to Kv1.x type channels, as 5-HT activation of 5-HT_2C_ receptors does not affect Kv3.1 channel activity (Aiyar et al., 1993). The exact cell signaling pathways underlying these effects remain unclear, but studies suggest 5-HT_2C_-Gα_q_-PLC signaling and activation of protein kinase C and tyrosine kinases PYK2 and Src are important contributors (Aiyar et al., 1993; Boland and Jackson, 1999; Imbrici et al., 2000; Speake et al., 2004).

Contrary to 5-HT_2C_ receptor's effects on Kv1.x channels, exposure to cocaine increases potassium currents, as measured in the NAc shell. Recent studies examining NAc shell GABAergic MSN show that these effects are mediated by an increase in the activity of Kv1.2 channels, leading to a decrease in excitability and firing rate (Hu et al., 2004; Kourrich and Thomas, 2009; Kourrich et al., 2013; Mu et al., 2010). Furthermore, exposure to cocaine for 10 consecutive days increases the expression of Kv1.2 channels (Kourrich et al., 2013). Importantly, the cocaine-induced depression of firing rate of MSN of the NAc shell is persistent, and decreases in the excitability of the NAc shell lead to enhanced locomotor responses and behavioral sensitization to cocaine and are associated with enhanced cocaine self-administration (Guillem et al., 2014; Kourrich and Thomas, 2009). Moreover, cocaine's inhibitory effects on MSN activity are specific to the NAc shell, with cocaine actually increasing MSN firing in the NAc core (Kourrich and Thomas, 2009; Kourrich et al., 2013; Mu et al., 2010). Finally, overexpression of a hyperpolarizing, inwardly-rectifying potassium channel (Kir2.1) in NAc MSN enhances psychomotor effects of cocaine (Dong et al., 2006), whereas knockdown of the Kv1.1 channel blocks behavioral effects of another psychostimulant, amphetamine (Ghelardini et al., 2003). Thus, there is a strong argument that some psychostimulant effects are mediated by alterations in intrinsic plasticity in NAc shell MSN, driven by Kv1.x currents that decrease excitability (Kourrich et al., 2015), and CH-mediated 5-HT_2C_ receptor activation may offset these effects (Figure 2).

## Caveat emptor

5-HT_2C_ receptors are expressed in several neural systems, including the frontal and cingulate cortices, that regulate impulsivity, approach, and reward behavior, and activation of these receptors impacts neural circuitry to modulate the effects of addictive drugs (Anastasio et al., 2014; Bubar and Cunningham, 2007; Daglish and Nutt, 2003; Liu et al., 2007; Nocjar et al., 2015). Our hypothesis regarding 5-HT_2C_ modulation of Kv1.x channels could be extended to these other neural systems. However, to maintain scope, we have focused on the NAc shell. Despite the logic regarding 5-HT_2C_ receptors and Kv1.x channels, there are reports that 5-HT_2A_ receptor activation also decreases Kv1.x activity, specifically Kv1.5 channels in cardiac tissue (Cogolludo et al., 2006) and Kv1.2 channels in cortical pyramidal neurons (Lambe and Aghajanian, 2001). In this latter study, 5-HT blocked Kv1.2 currents in layer V pyramidal neurons; the authors concluded the effects were mediated by 5-HT_2A_ receptors because of their dense expression. Still, 5-HT_2C_ receptors are also expressed in the frontal cortex, and modulate excitatory activity there (Beique et al., 2007). We acknowledge and appreciate that the modulatory activity of 5-HT_2_ receptor subtypes that affects behavior is complex. 5-HT_2_ receptor subtypes may modulate activity of unique Kv1.x channels, and the discovery of 5-HT_2_ receptor subtype expression patterns within discrete neural circuits will likely lead to further elucidation of their individual functions. For example, 5-HT_2A_ receptors are found in the NAc shell and core (Lopez-Gimenez et al., 2002; Mijnster et al., 1997), but (like 5-HT_2C_) the functions of 5-HT_2A_ receptors in subregions of the NAc are unclear. Additional studies are needed to cull the specific Kv1.x channel subtypes that may associate with 5-HT_2C_ to lead to suppression of addictive behaviors.

Many cell types and many unique afferents are found within the NAc and VTA (Russo and Nestler, 2013). Thus, a complete picture must show which cells express 5-HT_2A_, 5-HT_2B_, and 5-HT_2C_ receptors, the relative densities of receptors, and their unique contributions to network activity. Finally, 5-HT_2C_ chemical probes, including both agonists and antagonists, are, with few exceptions, notoriously non-selective (Canal and Morgan, 2012; Cussac et al., 2002; Porter et al., 1999). We have found, for example, that the “selective” 5-HT_2C_ agonist, Ro 60-0175, is a potent and efficacious 5-HT_2A_ agonist at the Gα_q_-PLC pathway, and when administered systemically at 3 mg/kg, Ro 60-0175 produces a HTR in C57BL/6J mice (unreported observations; data available upon request). It would be interesting to compare the behavioral and neurochemical effects of Ro 60-0175 directly to the more selective 5-HT_2C_ agonist lorcaserin, as well as to the recently reported selective 5-HT_2A_ agonist 25CN-NBOH (Fantegrossi et al., 2015; Hansen et al., 2014) to tease apart contributions of the 5-HT_2_ receptor subtypes more clearly (see the section on testable hypotheses below).

## Classic hallucinogens treat addiction

Literature from the 1950s through the 1970s, as well as modern reports, show that CH have positive effects on substance dependence (Bogenschutz and Johnson, 2016; Bogenschutz and Pommy, 2012; Dyck, 2006). Thus, not only do CH appear to be non-addictive, several studies show that they reverse the addictive effects of other psychoactive drugs. CH have been used to alleviate neurotic symptoms by indigenous people for thousands of years (Grinspoon and Bakalar, 1986), and the discovery of LSD and its psychoactive effects sparked considerable interest in the therapeutic potential of CH. Most work focused on the use of these compounds as an adjunct to psychotherapy. Between 1950 and the mid-1960s, more than 1000 quasi-clinical trials were completed, several dozen books were published, and six international conferences were held providing data on approximately 40,000 patients that had undergone “psychedelic” therapy sessions (Grinspoon and Bakalar, 1986; Riedlinger and Riedlinger, 1994). In the context of the treatment of substance dependence, most studies focused on the use of LSD for the treatment of alcoholism (for reviews, see Abuzzahab and Anderson, 1971; Bogenschutz and Johnson, 2016; Dyck, 2005; Grinspoon and Bakalar, 1986; Mangini, 1998). These studies examined both single, high-dose, and repeated low-dose designs, follow-up periods of several years, control groups, and a total subject pool of well in excessive of 1000 individuals (Abuzzahab and Anderson, 1971). Between 50% and 70% of the subjects showed reduced drinking or sobriety and/or improved social or professional functioning. Such a treatment effect is exceptional compared to current Food and Drug Administration–approved medications for alcoholism (i.e., disulfiram, naltrexone, and acamprosate). However, the relatively low number of participants in each study, lax designs, and suspect claims led many to question whether accurate conclusions could be made. Nevertheless, results from the six randomized trials of LSD for alcohol dependence that reported drinking outcomes (Bowen et al., 1970; Hollister et al., 1969; Ludwig et al., 1969; Pahnke et al., 1970; Smart et al., 1966; Tomsovic and Edwards, 1970) recently underwent a meta-analysis (Krebs and Johansen, 2012) that demonstrated consistent treatment effects supporting the efficacy of LSD.

Work with CH largely terminated by the 1970s due to the governmental regulatory response to advocates of the use of these drugs outside of the medical arena. Nonetheless, in the last decade, there has been a resurgence in the study of CH in general, and in the use of CH for the treatment of substance dependence in particular. This recent work has foregone the use of LSD in favor of psilocybin, and has extended our knowledge of the beneficial effects of CH to nicotine dependence (Bogenschutz et al., 2015; Johnson et al., 2014). We note that some researchers appreciate that stimulation of 5-HT_2A_ receptors (which are generally what come to mind when people think of CH) may exacerbate substance dependence (as discussed above) and have speculated that profound, rapid, and long-lasting agonist stimulated downregulation of 5-HT_2A_ receptors (i.e., functional antagonism) could be responsible for the anti-addiction effects of CH (Bogenschutz and Johnson, 2016).

Herein, we have proposed a non-exclusive and empirically testable hypothesis that the beneficial effects of CH for substance dependence are mediated by agonist stimulation of 5-HT_2C_ receptors. If, however, CH were both to stimulate 5-HT_2C_ receptors acutely and result in long-term downregulation of 5-HT_2A_ receptors, this may account for their purported therapeutic effects. Indeed, if CH treat addiction simply by stimulating 5-HT_2C_ receptors, it would likely be preferable to use a selective 5-HT_2C_ receptor agonist instead of CH. Although, others surmise that CH may be effective against addiction because of their psychedelic effects. They can elicit peak or mystical experiences, and afterglow effects, which can alter engrained personality domains such as openness (Maclean et al., 2011; Majic et al., 2015). These effects may be beneficial for psychotherapy and loosening relatively fixed behavioral patterns that underlie addiction.

Despite the potential for CH to treat addiction, it is important to caution that repeated exposure to CH could also chronically alter 5-HT_2C_ receptor expression and/or function, potentially leading to an enhanced susceptibility to addiction via a loss of intrinsic efficacy of 5-HT_2C_ receptors to modulate reward circuitry. Indeed, it does not escape our awareness that use of CH does not render persons immune to drug addiction, and a recent report shows that psilocybin users were significantly more likely to use addictive drugs (Hallock et al., 2013). Thus, medical supervision, psychological support, and careful preparation to control for set and setting in the context of CH use are likely important requirements to produce therapeutic effects.

## Testable hypotheses for unraveling the anti-addictive effects of classic hallucinogens

The literature we have reviewed above suggests a number of experiments that warrant testing in future studies. These experiments would serve to delineate more clearly the role of 5-HT_2_ receptor subtypes underlying the low addiction liability of CH. Morever, they would elucidate our understanding of the mechanisms involved, and the results would provide new testable hypotheses. Among these experiments are: (1) to determine whether recently synthesized compounds such as 25CN-NBOH, which shows ∼90-fold higher selectivity for activating 5-HT_2A_ over 5-HT_2C_, also show higher abuse liabilities than CH (caveat: that first the pharmacology of 25CN-NBOH is assessed at off-targets, e.g., opioid receptors, which would confound conclusions); (2) to determine whether animals with genetic deletion of the 5-HT_2C_ receptor will self-administer CH; (3) to determine the abuse liability of CH in the presence of a selective 5-HT_2C_ receptor antagonist; (4) to determine the precise localization of 5-HT_2C_ receptors in cells of the NAc, with particular emphasis on whether 5-HT_2C_ receptors are expressed on D1-expressing GABAergic MSN of the NAc shell (Caine et al., 2007; Lobo et al., 2010; Pacheco-Cano et al., 1996; Pisanu et al., 2015)—these MSN are the predominant MSN afferents of the VTA that modulate dopamine release in the NAc (Bocklisch et al., 2013); and (5) to determine the nature of 5-HT_2C_-Kv1.x interactions in forebrain regions implicated in addiction. We believe that these experiments would add greatly to the literature, and are enthused to see them completed.
